# Impact of sodium glucose cotransporter 2 (SGLT2) inhibitors on atherosclerosis: from pharmacology to pre-clinical and clinical therapeutics

**DOI:** 10.7150/thno.54498

**Published:** 2021-03-04

**Authors:** Zhenghong Liu, Xiaoxuan Ma, Iqra Ilyas, Xueying Zheng, Sihui Luo, Peter J. Little, Danielle Kamato, Amirhossein Sahebkar, Weiming Wu, Jianping Weng, Suowen Xu

**Affiliations:** 1 Department of Endocrinology, The First Affiliated Hospital of USTC, Division of Life Sciences and Medicine, University of Science and Technology of China, Hefei, China.; 2 Sunshine Coast Health Institute, University of the Sunshine Coast, Birtinya, QLD 4575, Australia.; 3 School of Pharmacy, Pharmacy Australia Centre of Excellence, the University of Queensland, Woolloongabba, Queensland 4102, Australia.; 4 Halal Research Center of IRI, FDA, Tehran, Iran.; 5 Biotechnology Research Center, Pharmaceutical Technology Institute, Mashhad University of Medical Sciences, Mashhad. Iran.; 6 Changshu Hospital Affiliated to Nanjing University of Chinese Medicine, Changshu, China.

**Keywords:** SGLT2 inhibitors, diabetes, atherosclerosis, therapy, cardiovascular complications

## Abstract

Sodium-glucose cotransporter 2 inhibitors (SGLT2i) are new oral drugs for the therapy of patients with type 2 diabetes mellitus (T2DM). Research in the past decade has shown that drugs of the SGLT2i class, such as empagliflozin, canagliflozin, and dapagliflozin, have pleiotropic effects in preventing cardiovascular diseases beyond their favorable impact on hyperglycemia. Of clinical relevance, recent landmark cardiovascular outcome trials have demonstrated that SGLT2i reduce major adverse cardiovascular events, hospitalization for heart failure, and cardiovascular death in T2DM patients with/without cardiovascular diseases (including atherosclerotic cardiovascular diseases and various types of heart failure). The major pharmacological action of SGLT2i is through inhibiting glucose re-absorption in the kidney and thus promoting glucose excretion. Studies in experimental models of atherosclerosis have shown that SGLT2i ameliorate the progression of atherosclerosis by mechanisms including inhibition of vascular inflammation, reduction in oxidative stress, reversing endothelial dysfunction, reducing foam cell formation and preventing platelet activation. Here, we summarize the anti-atherosclerotic actions and mechanisms of action of SGLT2i, with an aim to emphasize the clinical utility of this class of agents in preventing the insidious cardiovascular complications accompanying diabetes.

## Introduction

Atherosclerosis is the major potential pathology of most cardiovascular disease (CVD), including myocardial infarction (MI), heart failure (HF), stroke, and peripheral arterial disease 1. CVDs are the leading cause of morbidity and mortality globally 1, 2. In patients with diabetes, CVDs are the majority cause of premature mortality. Atherosclerosis is a slow-progressing inflammatory disease with a complex biochemical and cellular etiology characterized by the deposition of modified lipids in the arteries, the development of lipid-laden atherosclerotic plaques and ultimately the rupture of the plaque which precipitates the lethal clinical event being a heart attack or stroke 3, 4. The conventional risk factors for atherosclerosis and its thrombotic complications include hypertension, obesity, smoking, dyslipidemia, depression, sedentary lifestyles and diabetes 1. In particular, it is difficult to separate the effects of diabetes from those of other atherogenic factors. Patients with type 2 diabetes mellitus (T2DM) have a higher risk of atherosclerosis and other complications compared with those without diabetes, and ~80 percent of mortality in individuals with T2DM is due to cardiovascular events 5-7.

Sodium-glucose cotransporter 2 inhibitors (SGLT2i) have been developed as hypoglycemic drugs that target SGLT2, the major glucose transporter in the kidney responsible for about 90 percent of glucose reabsorption from primary urine 8. Recent evidence has suggested the use of SGLT2i as an adjunct to standard treatment to improve clinically relevant renal and cardiovascular outcomes in patients with T2DM 6, 9. SGLT2i can reduce glycosylated hemoglobin, body weight, blood pressure, plasma volume, increase in erythrocyte mass, and improve cardiac energy metabolism, which imposes a positive influence on cardiovascular risk factors and outcomes 5, 10-12. In light of the important clinical benefits of SGLT2i in improving cardiovascular outcomes, we provide a comprehensive and insightful overview of the pharmacological effects and underlying mechanisms of action of SGLT2i in CVD prevention, with a focus on mechanism addressing the accelerated atherosclerosis associated with diabetes.

## The pharmacological basis of SGLT2i

The SGLT2 protein, encoded by *SLC5A2*, is a member of the sodium-glucose cotransporter family and it undertakes the function of transporting glucose from the renal tubule lumen to renal tubule epithelial cells 13. SGLT2 is abundantly expressed in the anterior part of the proximal tubule 14, 15. Mining of GTEx database indicated that SGLT2 and SGLT1 are expressed in the kidney and intestine, respectively (Figure S1).

Phlorizin, the first natural SGLT2i, was isolated from the root bark of apple trees in 1835. Due to its low water solubility and poor absorption in the gastrointestinal tract, it was not developed as an anti-hyperglycemic agent 16. T-1095, a phlorizin derivative, overcomes some shortcomings of phlorizin, but could not go through clinical development 17. Later, c-aryl glycosides derived from the basic structure of phlorizin were subsequently developed, such as dapagliflozin and canagliflozin 18, 19. In addition to structural differences, they also have variable selectivity to SGLT1 and SGLT2 (Table 1) 20.

The expression of SGLT2 is up-regulated, and the urinary glucose excretion threshold is also higher in patients with hyperglycemia compared with healthy humans 21. Inhibition of SGLT2 reduces glucose reabsorption, promotes urinary glucose excretion, and produces negative caloric balance, which leads to weight loss 22. SGLT2i, including canagliflozin, dapagliflozin and empagliflozin, directly target SGLT2 instead of insulin secretion and insulin action as compared with other anti-hyperglycemic agents 13. SGLT2i can thus be used on top of other oral glucose-lowering drugs and insulin to exert additive anti-hyperglycemic effects 14.

At present, there are four SGLT2i (empagliflozin, canagliflozin, dapagliflozin, ertugliflozin) approved by the US Food and Drug Administration (FDA) and the European Union 23, 24. Some other drugs in the class like ipragliflozin, tofogliflozin and luseogliflozin are approved in Japan 25-27.

## Effects of SGLT2 inhibitors in CVD: clinical evidence

Many clinical studies have consistently shown that SGLT2i have multiple cardioprotective functions which manifest as reduced CVD (Table 2). The landmark EMPA-REG OUTCOME study was the first to offer convincing evidence that anti-diabetic drugs can reduce the occurrence of cardiovascular events. The trial randomly selected 7,020 diabetic patients with CVD. 3-point MACE (major adverse cardiovascular events, including death from cardiovascular causes, nonfatal MI, and nonfatal stroke), the primary outcome, was reduced by 14%. Hospitalization for heart failure (HHF) was reduced by 35% 9. However, the incidence of MI or stroke was not significant. The primary outcome is largely due to the reduction in death from cardiovascular causes 9. Subsequent experiments on renal effects of empagliflozin treatment also demonstrated that, compared with placebo, empagliflozin treatment group showed a slower progression of renal disease 28. Moreover, at 2020 at the European Society of Cardiology (ESC) annual meeting, the results of the EMPEROR-Reduced study, which extended the benefits of SGLT2i to patients with more advanced and severe chronic HF. The combined risk of HHF or cardiovascular death in patients receiving empagliflozin was 25% lower than placebo. In addition, empagliflozin-treated patients had a lower risk of serious renal outcomes 29, 30. In secondary analysis of the EMPEROR-Reduced trial indicated that patients treated with empagliflozin had improvement in health status 31. The efficacy and safety of empagliflozin in patients was not influenced by basal therapy with a neprilysin inhibitor 32. Combined treatment with both drugs may produce additional benefits 32.

Similar to EMPA-REG OUTCOME study, the CANVAS program showed a statistically significant reduction in 3-point MACE and HHF in the canagliflozin-treated patients 33. However, no benefit for non-fatal MI and stroke was observed. The composite renal endpoints were reduced by 27% for patients with canagliflozin therapy 33. Similarly, the CREDENCE trial analyzed cardiovascular, renal, and safety outcomes and showed that canagliflozin treatment reduced 3-point MACE and the stand-alone endpoint of HHF, as well as the risk of the primary outcome (end-stage kidney disease, doubling of serum creatinine, renal or cardiovascular death). This study also supported the concept that drugs of the SGLT2i class have clinical efficacy regardless of patients' HbA1c levels 34, 35.

The DECLARE-TIMI 58 trial indicated that treatment with dapagliflozin did not impact the 3-point MACE but significantly reduced the risk of HHF. Dapagliflozin also reduced the composite renal endpoint by 24 % 36, 37. Another trial, DAPA-HF, has shown that among patients with HF and reduced ejection fraction, patients receiving dapagliflozin had a lower risk of exacerbating HF or cardiovascular death than patients receiving placebo, regardless of whether they have diabetes or not 38. A *post hoc* analysis indicated that SGLT2i acted on background therapies of HF and reduced ejection fraction in a mechanistically-independent and complementary manner 39.

The VERTIS-CV study included 8,246 T2DM patients with confirmed disorder in coronary artery, cerebral and/or peripheral arterial system. The incidences of 3-point MACE in the ertugliflozin and placebo groups were similar, showing no significant difference, but ertugliflozin significantly reduced the risk of HHF 40. Patients who used SGLT2i had a lower risk of ischemic heart disease than those who did not use SGLT2i. The decrease in systolic blood pressure caused by SGLT2i was partially responsible for the results observed 41.

Despite the above-mentioned clinical trials showing the reduction in cardiovascular events in the SGLT2i treated groups (compared to placebo), only empagliflozin and canagliflozin had protective effects on 3-point MACEs 42. The favorable clinical outcomes are hypothesized to be mainly driven by reduction of the rate of HHF. However, some large multi-national observational studies in patients with T2DM and cardiovascular risk suggested beneficial effects of SGLT2i also directed to MI and stroke which are events most closely associated with atherosclerosis and its clinical sequalae 43, 44.

In contrast, another analysis found that glucose-lowering drugs including SGLT2i significantly reduced the risk of atherosclerotic events but had no significant effect on the risk of HF, indicating the need for further clinical and basic studies in this exciting new area of the therapeutics of diabetes and its CVD consequences 45.

A meta-analysis of three SGLT2i related clinical trials found that the reduction in 3-point MACE was not large, and this effect was limited to patients with established ASCVD 46. Also, the UTOPIA trial investigated the effects of tofogliflozin in T2DM patients without apparent CVD and indicated that tofogliflozin treatment did not delay the progression of atherosclerosis by monitoring carotid intima-media thickness but lowered arterial stiffness by evaluating the changes in brachial-ankle pulse wave velocity 47-49. This might be due to limited sample size and study duration 47. Therefore, increasing the sample size and research duration may provide some clues for whether or not these drugs have an influence on the progression of atherosclerosis.

## Effects of SGLT2 inhibitors on atherosclerosis: experimental evidence

Based on the notable cardiovascular benefits conferred by SGLT2i, research interest has been focused on the study of the anti-atherosclerotic effects of SGLT2i in suitable experimental models and several SGLT2i have been shown to ameliorate atherosclerosis in *ApoE^-/-^* mice, *Ldlr^-/-^*mice and rabbits (Table 3).

In the *ApoE^-/-^* mouse model, canagliflozin alleviated atherosclerosis by reducing the expression of monocyte chemoattractant protein-1 (MCP-1) and vascular cell adhesion molecule-1 (VCAM-1), accompanied by decreased levels of total cholesterol, triglyceride and glucose, and it also decreased heart rate, plaque size, and increased plaque stability 50. Canagliflozin also suppressed lipid synthesis and interleukin (IL)-1β levels in *ApoE^-/-^* mice 51. Similarly, luseogliflozin treatment inhibited the expression of intercellular cell adhesion molecule-1 (ICAM-1), IL-1β, IL-6, and tumor necrosis factor-α (TNF-α) 52. Luseogliflozin treatment reduced macrophage accumulation in perivascular adipose tissue and reduced neointimal hyperplasia 53. Ipragliflozin exerted similar actions (suppressed macrophage accumulation, reduced fibrosis and adipocyte death) 54. Empagliflozin reduced the levels of CD68, MCP-1, ICAM-1, TNF-α and nicotinamide adenine dinucleotide phosphate (NADPH) oxidase subunits and thereby ameliorated diabetes-induced endothelial dysfunction 55. Moreover, several studies have indicated that empagliflozin increased tissue inhibitor of metalloproteinase (TIMP)/matrix metalloproteinase-2 (MMP-2) ratio and increased collagen content of developing plaques, rendering the plaques more stable 50, 56. After empagliflozin treatment, the atherosclerotic plaque area was smaller, and the inflammatory cell infiltration in adipose tissue was reduced 57. A further study indicated that empagliflozin reduced angiotensin II-induced neovessel formation and macrophage infiltration in the abdominal aortic aneurysm lesions in* ApoE^-/-^* mice 58. In addition, empagliflozin treatment also exerted atheroprotection by inhibiting the renin-angiotensin-aldosterone system and sympathetic activity 59. In hyperglycemic STZ-diabetic mice, empagliflozin also reduced atherosclerotic plaques 60. Another study used *ApoE^-/-^* mice as a model of non-proteinuric diabetic kidney disease and found that empagliflozin treatment inhibited the development of aortic atherosclerosis and increased ketone body levels 61. Moreover, dapagliflozin treatment attenuated atherosclerosis, reduced macrophage infiltration, and enhanced plaque stability 62, 63. Similar results were obtained after ipragliflozin treatment 62. However, a study in *ApoE^-/-^Irs2^+/-^* mice indicated that dapagliflozin did not protect against the development of atherosclerosis in insulin-resistant mice under hypercholesterolemic conditions 64.

As dyslipidemia is an independent risk factor for atherosclerosis 65, it is also important to study how glycemic control affects the development of atherosclerosis in the presence of hyperlipidemia. Effective glycemic control with dapagliflozin not only reduced atherosclerosis, but also ameliorated plasma lipoprotein profiles in *Ldlr^-/-^* mice 66. The benefits of dapagliflozin on atherosclerosis have also been demonstrated in experimental animals other than mice. For example, in a rabbit model of atherosclerosis, dapagliflozin was found to exhibit anti-atherosclerotic effects by modulating inflammatory responses (decreased expression of TNF-α, IL-1β, and IL-6) and macrophage polarization (toward M2 macrophages) under non-diabetic conditions 67.

## Mechanisms of action of SGLT2 inhibitors

**Figure 1:**
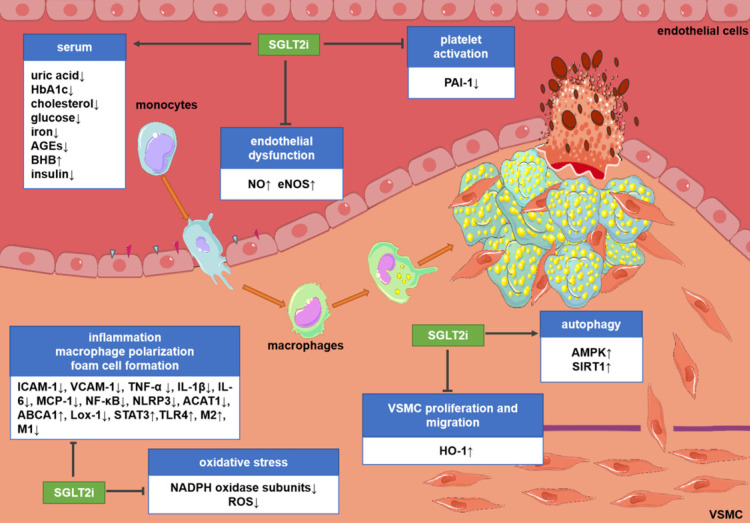
** Potential molecular targets of SGLT2i in atherosclerosis.** Although the existing evidence is not sufficient to directly prove the anti-atherosclerotic mechanism of action of SGLT2i, some preclinical and clinical studies have revealed some potential mechanisms. SGLT2i may inhibit the progression of atherosclerosis by impacting the levels of related inflammatory factors in the serum, inhibiting endothelial dysfunction, VSMC proliferation and migration, macrophage inflammation, foam cell formation, platelet activation, and oxidative stress and improve autophagy impairment. Abbreviations: ABCA1: ATP-binding cassette transporter A1; ACAT1: acetyl-coenzyme A acetyltransferase 1; AGEs: advanced glycation end-products; AMPK: AMP-activated protein kinase; BHB: β-hydroxybutyrate; HbA1c: glycosylated hemoglobin; eNOS; endothelial nitric oxide synthases; HO-1: hemeoxygenase-1; ICAM-1: intercellular cell adhesion molecule-1; IL-1β: interleukin-1β; IL-6: interleukin-6; Lox-1; lectin-like oxidized low-density lipoprotein receptor-1; M1: M1 macrophages; M2: M2 macrophages; MCP-1: monocyte chemoattractant protein-1; NADPH: nicotinamide adenine dinucleotide phosphate; NF-κB: nuclear factor-κB; NLRP3: nucleotide-binding domain-like receptor protein 3; NO: nitric oxide; PAI-1: plasminogen activator inhibitor-1; ROS: reactive oxygen species; SIRT1: sirtuin-1; STAT3: signal transducer and activator of transcription 3; TLR4: toll-like receptors; TNF-α: tumor necrosis factor-α; VCAM-1: vascular cell adhesion molecule-1.

**Figure 2:**
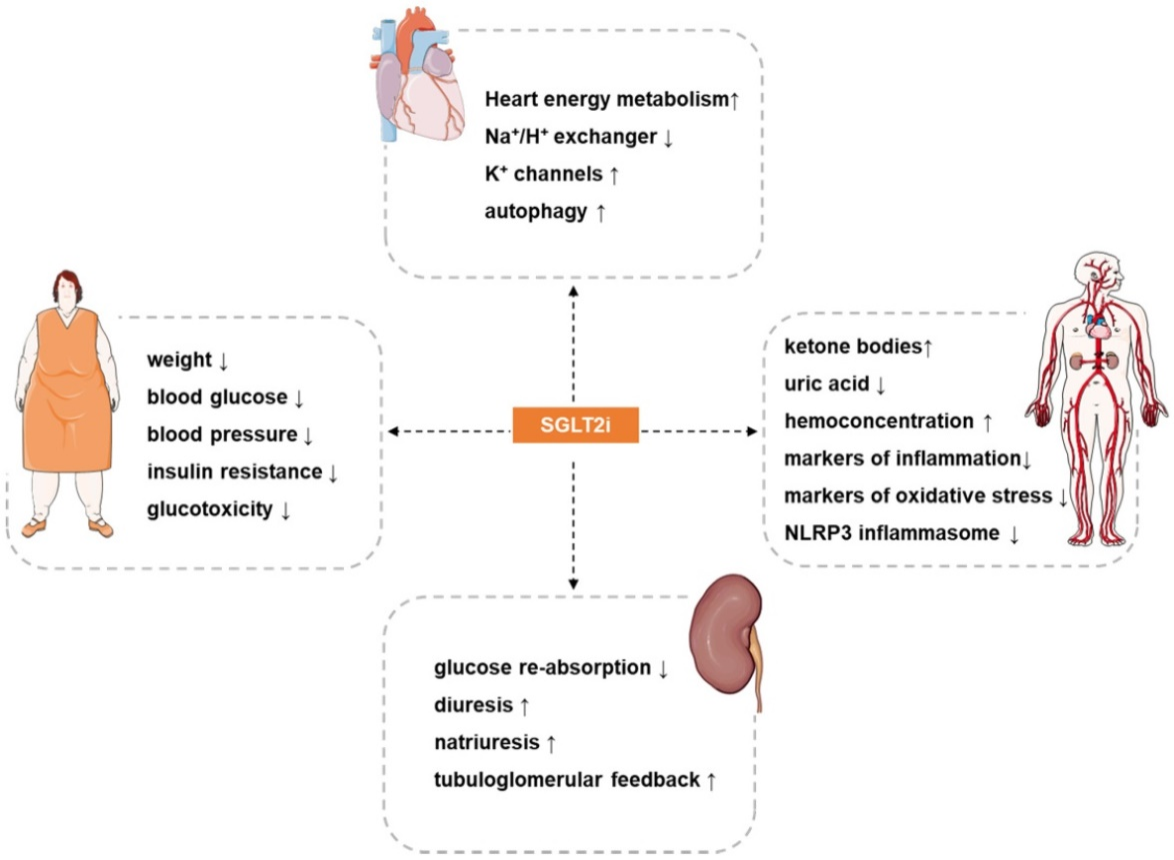
** Potential cardiovascular actions of SGLT2i.** SGLT2i have pleiotropic cardiovascular protective effects, such as: reduce weight, blood pressure, blood glucose, insulin resistance and glucotoxicity in patients, increases hemoconcentration, and inhibits oxidative stress and inflammation. The most direct effect of SGLT2i is inhibition of the reabsorption of glucose and a diuretic and natriuretic effect. In addition, SGLT2i also exerts other effects such as regulating ion channels, activating autophagy, inhibiting iron overload, attenuating activation of the NLRP3 inflammasome, and inhibiting the signaling of advanced glycation end products. The synergistic effects of these benefits may provide a therapeutic basis for the cardioprotective effects of SGLT2i.

The main mechanism for SGLT2i to exert hypoglycemic effects is to increase the excretion of glucose in urine 10, 68. However, in diabetic patients, the mechanism of the inhibitory effect of SGLT2i on atherosclerosis, which is the cause of cardiovascular events, remains unclear. The focus of clinical trials is to study the impact of SGLT2i on cardiovascular events, deaths and safety outcomes, but the research on their mechanism is mainly based on preclinical studies. In the past decade, various targets and signaling pathways mediating SGLT2i's cardioprotective actions have been revealed. The potential molecular targets and beneficial effects of SGLT2i on atherosclerosis are discussed as below (Figure 1 and Figure 2).

### Improving endothelial dysfunction

Endothelial dysfunction is an initial key event of atherosclerosis and an important contributor to vascular diseases 69, 70. Substantial evidence showed that SGLT2i ameliorate endothelial dysfunction and improve endothelium-dependent vasodilation. Dapagliflozin regulated glycemic indices, which could improve flow-mediated vasodilation, arterial stiffness and endothelial function in patients with T2DM 71-73.

Several preclinical studies have demonstrated that endothelial dysfunction can be prevented by SGLT2i in different experimental models. Empagliflozin prevented the increased expression of atherothrombotic markers and improved endothelial function in ZSF1 rats that have metabolic syndrome and associated insulin resistance 74. In this context, empagliflozin treatment decreased aortic stiffness and suppressed endothelial dysfunction by promoting glycosuria in a mouse model of T2DM 75. Furthermore, empagliflozin attenuated high glucose-induced endothelial senescence and dysfunction by inhibiting the local angiotensin system 76. Similarly, dapagliflozin reduced arterial stiffness and endothelial dysfunction in diabetic mice and enhanced diastolic function in a non-diabetic model 77, 78. SGLT2i reversed endothelial activation and endothelial nitric oxide synthases (eNOS) deficit under diabetic conditions 78. These results are consistent with those of Gaspari *et al*. 79, who found that dapagliflozin treatment attenuated vascular endothelial cell activation and induced significant endothelium-independent vasorelaxation in *ApoE^-/-^* mice. Importantly, Tahara *et al*. 80 conducted a comparative experiment to compare the effects of six SGLT2i (luseogliflozin, ipragliflozin, tofogliflozin, empagliflozin, canagliflozin and dapagliflozin) on diabetes-related complications in T2DM mice and determined that all SGLT2i examined prevented the development of endothelial dysfunction suggesting this is a class effect for these agents although the commonality of reduced glycemia cannot be totally excluded.

### Improving vascular smooth muscle cell dysfunction

Excessive proliferation and migration of vascular smooth muscle cells (VSMCs) as part of the development of the neointima play a crucial role in the pathogenesis of atherosclerosis 81, 82. In this regard, VSMC growth and migration were significantly blunted in diabetic patients after canagliflozin treatment at clinically relevant doses 83. Heme oxygenase-1 (HO-1) is a newly discovered target of canagliflozin. Treatment of VSMCs with canagliflozin stimulated HO-1 expression/activity 83.

Combination therapy with ipragliflozin and empagliflozin inhibited VSMC proliferation and the formation of neointima after vascular injury 84. Furthermore, empagliflozin improved coronary microvascular function and contractile function 85. Ipragliflozin also had the same actions (inhibiting the proliferation and migration of monocytes and VSMCs *in vitro*) 54.

### Attenuation of macrophage inflammation, foam cell formation, and M1 polarization

Macrophage inflammation, foam cell formation, and M1 polarization are critical events in the development of atherosclerosis 86. Empagliflozin ameliorated cardiac macrophage infiltration in db/db mice 87. Similar findings were reported by Pennig *et al.* 60 using STZ-induced diabetic mice. Glucose-lowering effects conferred by empagliflozin alleviated the proliferation of plaque-resident macrophages and the atherosclerotic plaque size was significantly smaller 60. In addition, mechanistic studies revealed that empagliflozin reduced the accumulation of M1 polarized macrophages, and redirected the macrophage phenotype toward an anti-inflammatory M2 phenotype, reduced obesity-related chronic inflammation, attenuated insulin resistance, and activated AMP-activated protein kinase (AMPK) 88, 89. Similarly, canagliflozin directly inhibited the secretion of endothelial pro-inflammatory cytokine (MCP-1 and IL-6) through AMPK-dependent and -independent mechanisms 90. AMPK activation increased ATP production and reduced ATP consumption 89. The expression of lectin-like oxidized low-density lipoprotein receptor-1 (Lox-1) and acetyl-coenzyme A acetyltransferase 1 (ACAT1) genes was down-regulated in peritoneal macrophages isolated from diabetic mice receiving dapagliflozin, while the expression of ATP-binding cassette transporter A1 (ABCA1) was up-regulated 63.

In addition, macrophage infiltration into atherosclerotic lesions was reduced by dapagliflozin treatment 63. In an infarction model in non-diabetic rats, dapagliflozin increased signal transducer and activator of transcription 3 (STAT3) activity, STAT3 nuclear translocation, and M2 macrophage infiltration. 91. Similar results were reported in a rabbit model, in which dapagliflozin increased M2 macrophages and inhibited toll-like receptor 4/nuclear factor-kappa B signaling pathway which serve as master regulators of inflammatory responses in macrophages 67.

### Prevention of platelet activation

Platelet adhesion, activation and aggregation in plaques are key events in atherothrombosis 92. The reduction in blood glucose by dapagliflozin treatment normalized reticulated platelet levels 93. Spigoni* et al.* 94 showed that empagliflozin and dapagliflozin reduced inflammation and oxidative stress and might reduce ADP-stimulated platelet activation. Empagliflozin reduced the plasma concentration of plasminogen activator inhibitor-1 in patients with T2DM, which inhibited the development of thrombotic diseases 95. Therefore, plaque stabilization and inhibition of thrombosis are the potential mechanisms of SGLT2i-mediated cardiovascular protection 94.

### Attenuation of oxidative stress

The development of atherosclerosis is closely related to oxidative stress. SGLT2i reduce oxidative stress in patients, experimental animals, and cultured cells. After SGLT2i treatment, NADPH oxidase subunits (NOX1, NOX2, NOX4, p22phox, and p47phox) were reduced 55, 96-98. Surrogate parameters of oxidative stress, 3-nitrotyrosine- and hydroxynonenal-positive proteins, were almost normalized 99. Moreover, parameters of pathological oxidative stress (hydrogen peroxide, 3-nitrotyrosine, lipid peroxide) were attenuated in cardiomyocytes 100 and urinary excretion of 8-hydroxydeoxyguanosine was reduced 97, 101. Inhibition of oxidative stress restores the bioavailability of NO and explains the vasoprotective benefits of SGLT2i 102. Kolijn *et al.* 100 conducted more in-depth mechanistic research and observed that empagliflozin improved endothelial vasorelaxation *via* reducing pro-inflammatory/pro-oxidative pathways and eNOS-dependent PKGIα (cyclic guanosine monophosphate-dependent protein kinase G Iα) oxidation. SGLT2i improved PAR2 (proteinase-activated receptor 2)-mediated NOS-dependent vasodilation, which is compromised by oxidative stress though an NAPDH oxidase/ROS-dependent signaling pathway 103.

### Reduced inflammation

Compared with most current glucose**-**lowering agents, SGLT2i have actions in reducing tissue inflammation. Evidence in mouse models suggested that SGLT2i inhibited the expression of circulating inflammatory molecules (TNF-α, MCP-1, PECAM-1, VCAM-1, ICAM-1, IL-1β, and IL-6) associated with atherosclerosis 52, 56, 62, 77. Also, human evidence indicated that canagliflozin might induce changes in TNFR1, IL-6, MMP7, serum leptin, adiponectin and fibronectin 1 104, 105. Empagliflozin reduced superoxide production in leukocytes and reduced hs-CRP in patients with T2DM 106. SGLT2i have the capacity to inhibit inflammation and reverse the adverse factors of atherosclerosis.

### Regulation of iron metabolism

Iron metabolism occurs as a complex interplay between iron *per se*, inflammation and atherosclerosis 107. Iron overload promotes the formation of highly reactive forms of oxygen free radicals, which accelerates atherosclerosis 108-111. Serum ferritin is a reliable indicator of iron stores 110, 112. High transferrin saturation signals iron overload 108. Recent proteomic findings in plasma of T2DM demonstrated significant decrement in ferritin following empagliflozin treatment 113. In addition, dapagliflozin treatment significantly reduced circulating hepcidin and ferritin concentrations 114. Regulating iron metabolism might be one of the novel mechanisms of action of SGLT2i in cardiovascular protection but this area requires more investigation.

### Promoting autophagy

Autophagy is related to the clearance of apoptotic macrophages from atherosclerotic plaques 115. Blocking autophagy renders macrophages more susceptible to cell death and promotes necrosis in advanced atherosclerosis 115. Canagliflozin inhibited intracellular glucose metabolism and promoted autophagy that might be associated with inhibited 6-phosphofructo-2-kinase (PFK2) expression and increased AMPK phosphorylation 116. Autophagy is closely related to AMPK and sirtuin-1 (SIRT1). Canagliflozin upregulated the expression of SIRT1 117. Similarly, empagliflozin treatment activated AMPK and enhanced cardiac autophagy 118. Following MI in patients with diabetes, empagliflozin inhibited ROS and restored autophagy to normalize the size and number of mitochondria 119. Empagliflozin treatment increased the level of mitochondrial SIRT3 and enhanced the activation of TLR9, thereby activating autophagy 120. Therefore, enhancing autophagy might be a potential mechanism for SGLT2i to exert atheroprotective effects.

### Regulation of ion exchange channels

K^+^ channels regulating depolarization/hyperpolarization are the main determinants of vascular tone. The voltage-dependent K^+^ (Kv) channels could be the target of dapagliflozin. The vasodilatory effect of dapagliflozin occured through direct activation of protein kinase G and subsequent activation of Kv channels 121.

Na^+^/H^+^ exchanger 1 (NHE1) in endothelial cells might be another target of SGLT2i. Dapagliflozin inhibited the activity of NHE1 in endothelial cells to reverse endothelial activation 78. Empagliflozin treatment directly inhibited NHE1 mediated Na^+^ influx, thereby reducing myocardial cytoplasmic Na^+^, regardless of SGLT2 activity 122. However, the latest research proves that empagliflozin treatment did not inhibit cardiac NHE1 activity 123. It remains unclear whether SGLT2i affect the progression of atherosclerosis through targeting ion channels.

### Increasing ketone bodies

An important feature of diabetic patients treated with SGLT2i is the increase of circulating ketone bodies 124. Ferrannini *et al*. 125-127 indicated that increased β-hydroxybutyrate (BHB) promote ketone bodies as metabolic substrates and result in improved energy metabolism of the heart. In addition to the involvement in energy metabolism, other protective effects have been proposed for ketone bodies. For example, preclinical findings demonstrate that BHB has a strong anti-inflammatory effect. Empagliflozin has been reported to significantly increase the abundance of serum BHB leading to inhibition of NLRP3 and reduction of IL-1β levels 128. The importance of ketone bodies as an adjuster of the benefits of SGLT2i in atherosclerosis remain uncertain.

### Reduced body weight

Inhibition of glucose reabsorption leads to calorie loss, accompanied by weight loss 129. Several meta-analyses of clinical trials in patients with T2DM have suggested that body weight was significantly reduced following SGLT2i treatment 130, 131. SGLT2i convert glucose metabolism into fatty acids and ketones, and enhance fat utilization that are favorable factors which confer anti-atherosclerotic effects.

### Regulation of diuresis, natriuresis, hemoconcentration and blood pressure

SGLT2i have natriuretic and diuretic effects 124. Induction of diuresis and natriuresis by SGLT2i decrease plasma volume and contribute to systolic and diastolic blood pressure control 132, 133. Hypertension is a contributing factor to atherosclerosis and its thrombotic complications 1. Reductions in blood pressure were greater with empagliflozin compared with placebo 134. Natriuresis also activates the tubuloglomerular feedback response 135. The synergistic effect of these several mechanisms may provide an indirect but useful basis for the anti-atherosclerotic effects of SGLT2i.

### Lowering the level of uric acid

SGLT2i treatment resulted in lower circulating levels of uric acid 136-138. Uric acid is considered an activator of oxidative stress and inflammation, which induces activation of the NLRP3 inflammasome 124, 139. Lowering uric acid might be an indirect mechanism of SGLT2i to improve atherosclerosis, and its deeper mechanism remains to be evaluated.

### Inhibition of NLRP3 inflammasome

Nucleotide-binding domain-like receptor protein 3 (NLRP3) inflammasome plays a vital role in inflammation and immunity 140. The activation of NLRP3 inflammasome and the subsequent release of IL-1β and IL-18 contribute to the pathogenesis of atherosclerosis and HF 141-143. Current research on the effect of SGLT2i on NLRP3 inflammasome is focused on diabetic nephropathy, steatohepatitis, cardiomyopathy and atherosclerosis.

Empagliflozin attenuated the activation of NLRP3 inflammasome in a Ca^2+^-dependent manner 144. Kim *et al.* 128 demonstrated that empagliflozin significantly inhibited the activation of NLRP3 inflammasome by increasing serum BHB levels and reducing insulin levels in T2DM and CVD patients, regardless of glycemic control. Dapagliflozin treatment reduced the production of NLRP3 protein and ROS in aortic tissues, thereby partially reversing the formation of atherosclerosis 62. Dapagliflozin also inhibited the activation of NLRP3 inflammasome by activating AMPK and mTORC2 145, 146. In conclusion, SGLT2i attenuates the activation of NLRP3 inflammasome, which might help explain its inhibitory effect on atherosclerosis.

### Reduction of advanced glycation end-products

The binding of advanced glycation end-products (AGEs) to endothelial AGE receptors (RAGE) stimulates oxidative stress and expression of cytokines, chemokines, and adhesion molecules 147. Methylglyoxal, a primary precursor of AGEs, decreased the phosphorylation of eNOS^Ser1177^ and protein kinase B (Akt), which inhibited eNOS activity. SGLT2i decreased the levels of methylglyoxal, prevented AGE formation and AGE/RAGE signaling, and ameliorated decreased phosphorylation of eNOS^Ser1177^ and Akt, thus conferring atheroprotective effects 96, 97, 101.

## Conclusions and perspectives

As a new category of oral hypoglycemic agents, SGLT2i have a specific mechanism of action and target glucose removal which is distinct from other hypoglycemic agents. By increasing the excretion of urinary glucose, SGLT2i regulate glucose levels without an increased risk of hypoglycemic events. A recent observational study suggested that SGLT2i might be more effective than GLP-1RA in ameliorating cardiovascular outcomes of T2DM with comparable rate of adverse events 148. In addition, SGLT2i significantly decreased the risk of HF or cardiovascular death independent of diabetes status in patients on background therapy for HF 39, 149.

The cardiovascular actions and anti-inflammatory effects of SGLT2i have been excellently reviewed elsewhere 11, 150-153. Here, we provide a focused review of the protective effects of SGLT2i in different stages of atherosclerosis (the leading cause of CVD), illuminating the molecular targets of this category of drugs in atheroprotection. In patients with diabetes, SGLT2i show cardio-renal protection and have important clinical advantages but there are also some adverse reactions. The most commonly observed adverse effect is polyuria. Empagliflozin increased the risk of urogenital infections in women and men 9. Another important safety concern, observed in the CANVAS trial, was amputations and fractures of the legs and feet in patients treated with canagliflozin compared with placebo 33. However, a recent real-world study suggested that the risk of amputations in patients treated with SGLT2i was not higher compared with other anti-diabetic drugs 154. Also, the application of SGLT2i for patients with type 1 diabetes should be considered with caution due to increased incidence of ketoacidosis and diarrhea 155. Long-term systemic side effects of SGLT2i are warranted to be evaluated in large-scale randomized controlled trials.

By deepened understanding of the mechanism of action of SGLT2i, the adverse reactions after drug treatments could be reduced. Results of recent clinical trials involving individuals without diabetes might repurpose this drug as “a drug for cardiorenal protection” 156. Taken together, SGLT2i have broad therapeutic prospects, and their pharmacological mechanisms and precise molecular targets beyond SGLT2 inhibition and glycemic control need to be elucidated in future studies.

## Supplementary Material

Supplementary figure.

## Figures and Tables

**Table 1:** Approved SGLT2 inhibitors in clinics

SGLT2i	Pubchem CID	Recommended starting dose (once daily)	Structure	selectivity (SGLT2:SGLT1)
Empagliflozin	11949646	10 mg	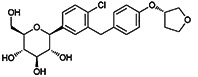	~ 2700:1
Dapagliflozin	9887712	5 mg	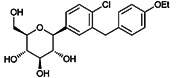	~ 1200:1
Canagliflozin	24812758	100 mg	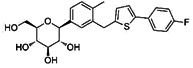	~ 414:1
Ipragliflozin	10453870	50 mg	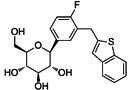	~ 860:1
Tofogliflozin	46908929	20 mg	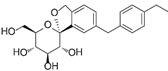	~ 3000:1
Luseogliflozin	11988953	2.5 mg	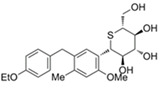	~ 1770:1
Ertugliflozin	44814423	5 mg	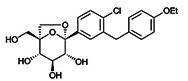	~ 2200:1

**Table 2:** Completed clinical trials of SGLT2i in patients with T2DM, CVD or both

Drugs	Trials	Patients	Median follow-up	Outcomes				References
				3-Point MACE	CV Death	HHF	CV Death or HHF	
Empagliflozin	EMPA-REG	7,020 T2DM patients with CVD.	3.1 years	0.86 (0.74-0.99) *	0.62 (0.49-0.77) *	0.65 (0.50-0.85) *	0.66 (0.55-0.79) *	9
	EMPEROR-Reduced	3,600 patients with HF and reduced ejection fraction (≤40%).	16 months	——	——	0.70 (0.58-0.85) *	0.75 (0.65-0.86) *	30
Canagliflozin	CANVAS	10,142 T2DM patients with CVD or CV risk factors.	2.4 years	0.86 (0.75-0.97) *	0.87 (0.72-1.06)	0.67 (0.52-0.87) *	——	33
	CREDENCE	4,401 T2DM patients with CKD.	2.6 years	0.80 (0.67-0.95) *	——	0.61 (0.47-0.80) *	——	35
Dapagliflozin	DECLARE-TIMI 58	17,160 T2DM patients with ASCVD or CV risk factors.	4.2 years	0.93 (0.84-1.03)	0.98 (0.82-1.17)	0.73 (0.61-0.88) *	0.83 (0.73-0.95)	36
	DAPA - HF	4,744 patients with HF and reduced ejection fraction.	18.2 months	——	0.82 (0.69-0.98)	0.70 (0.59-0.83)	0.75 (0.65-0.85) *	38
Ertugliflozin	VERTIS-CV	8,246 T2DM patients with ASCVD.	3.5 years	0.97 (0.85-1.11)	0.92 (0.77-1.11)	0.70 (0.54-0.90)	——	40

ASCVD: atherosclerotic cardiovascular diseases; CKD: chronic kidney disease; CV: cardiovascular; CVD: cardiovascular diseases; HF: heart failure; HHF: hospitalization for heart failure; MACE: major adverse cardiovascular events; T2DM: type 2 diabetes mellitus; "*" statistically significant difference.

**Table 3:** Atheroprotective effects and mechanisms of SGLT2i in rodents

Drugs	Animal model	Treatment dose and duration	Observations and mechanisms	References
Empagliflozin	*ApoE*^-/-^ mice with HFD containing 0.2% cholesterol	10 mg/kg/day for 10 weeks *via* oral gavage	atherosclerosis↓, total cholesterol, fasting glucose↓, heart rate diastolic, blood pressure↓ , VCAM-1, MCP-1↓	56
Empagliflozin	*ApoE*^-/-^ mice with STZ-induced diabetes and western type diet	20 mg/kg/day for 12 or 8 weeks *via* oral gavage	atherosclerosis↓, endothelial dysfunction↓, plasma triglyceride↓, CD68, MCP-1, TNF-α, ICAM-1↓, NADPH oxidase subunits ↓, vasoconstrictive eicosanoids ↓, prostaglandin E2, thromboxane B2 ↓	55
Empagliflozin	*ApoE*^-/-^ mice with western diet containing cholesterol	1 mg/kg or 3 mg/kg for 10 weeks *via* oral	atherosclerosis↓, TNF-α, IL-6, MCP-1, CD68↓, serum amyloid A, urinary microalbumin↓	57
Empagliflozin	Ang II-infused *ApoE*^-/-^ mice	1 mg/kg/day or 3 mg/kg/day for 4 weeks *via* oral gavage	abdominal aortic aneurysm ↓, elastin degradation, neovessel formation, macrophage infiltration↓, CCL-2, CCL-5, VEGF↓, MMP-2, MMP-9↓, p38 MAPK, NF-κB↓	58
Empagliflozin	*ApoE*^-/-^ mice with HFD	30 mg/kg/day for 8 weeks *via* oral gavage	atherosclerosis↓, endogenous ketone body↑, mTORC1↓	61
Empagliflozin	STZ-diabetic mice with injections of LDLR and SRB1 antisense oligonucleotides and high -cholesterol diet (HCD) for 16 weeks	35 mg/kg/day for 3 weeks *via* drinking water	atherosclerosis↓, lipid↓, CD68↓	60
Empagliflozin	*ApoE*^-/-^ mice with western diet containing 0.2 % cholesterol	10 mg/kg/day for 5 weeks *via* drinking water	atherosclerosis↓, triglyceride, total cholesterol, LDL↓, the renin-angiotensin-aldosterone system and sympathetic activity↓, body weight↓	59
Dapagliflozin	*ApoE*^-/-^ mice with HFD and STZ-induced diabetes	1.0 mg/kg/day for 12 weeks *via* gavage	atherosclerosis↓, macrophage infiltration↓, smooth muscle cell proliferation↓, fasting glucose ↓, cholesterol crystals ↓, IL-1β, IL-18, NLRP3, ROS↓, ROS-NLRP3-caspase-1 pathway.	62
Dapagliflozin	*ApoE^-/-^Irs2^+/-^* mice with a high-fat, high-cholesterol diet	3 mg/kg/day for 6 weeks	No effect on circulating inflammatory cells or cytokine level, no protection against atherosclerosis.	64
Dapagliflozin	*Ldlr*^-/-^ mice with STZ- induced diabetes and 0.15% cholesterol diet	25 mg/kg for 4 weeks *via* drinking water	atherosclerosis↓, plasma glucose, total cholesterol, triglycerides↓, lipoprotein clearance↑, HSPG and bile acid pathways.	66
Dapagliflozin	Rabbit with 1% high-cholesterol diet and balloon injury in aorta	1 mg/kg/day for 8 weeks	atherosclerosis↓, macrophage infiltration↓, TNF-α, IL-1β, IL-6↓, M2 macrophages↑	67
Canagliflozin	*ApoE*^-/-^ mice with HFD containing 0.2% cholesterol	10 mg/kg/day for 5 weeks *via* oral	atherosclerosis↓, total cholesterol, triglycerides↓, VCAM‑1, MCP‑1↓, TIMP‑1/MMP‑2↑	50
Canagliflozin	*ApoE*^-/-^ mice with HFD containing 0.2% cholesterol	30 mg/kg/day for 4 weeks *via* oral gavage	energy expenditure↑, adiposity↓, liver lipid synthesis↓, IL-1β↓	51
Ipragliflozin	wild-type mice with Western-type diet	10 mg/kg/day for 10 weeks *via* drinking water	macrophages accumulation, fibrosis, and adipocyte death↓ monocytes and VSMCs migration↓	54
Dapagliflozin or Ipragliflozin	*ApoE*^-/-^ mice with STZ-induced diabetes and atherogenic diet	1.0 mg/kg/day for 4 weeks *via* drinking water	atherosclerosis ↓, macrophage infiltration↓, foam cell formation↓, HbA1c↓, ABCA1↑ACAT1↓	63
Luseogliflozin	*ApoE*^-/-^ mice with NA- and STZ- induced diabetes	dose with maximal glucose-lowering efficacy for 1 week *via* diet	atherosclerosis↓, F4/80, TNFα, IL-1β, IL-6↓, ICAM-1, PECAM-1, MMP2, MMP9↓	52
Luseogliflozin	Wild‑type mice fed with low‑fat diet or HFD	18 mg/kg/day for 25 days *via* diet	adipocyte sizes↓, accumulation of macrophages expressing PDGF-B↓, adiponectin gene expression↑	53

ABCA1: ATP-binding cassette transporter A1; ACAT1: acetyl-coenzyme A acetyltransferase 1; CCL: chemokine (C-C motif) ligand; HbA1c: glycosylated hemoglobin; HFD: high‑fat diet, HSPG: heparan sulfate proteoglycans; ICAM-1: intercellular cell adhesion molecule-1; LDLR: low-density lipoprotein receptor; IL-1β: interleukin-1β; IL-6: interleukin-6; IL-18: interleukin-18; MAPK: mitogen-activated protein kinase; MCP-1: monocyte chemoattractant protein-1; MMP: matrix metalloproteinase; NADPH: nicotinamide adenine dinucleotide phosphate; NF-κB: nuclear factor-κB; NLRP3: nucleotide-binding domain-like receptor protein 3; PDGF-B: platelet‑derived growth factor‑B; PECAM-1: platelet endothelial cell adhesion molecule-1; ROS: reactive oxygen species; SRB1: scavenger receptor B1; TIMP: tissue inhibitor of metalloproteinase; TNF-α: tumor necrosis factor-α; VCAM-1: vascular cell adhesion molecule-1; VEGF: vascular endothelial growth factor; VSMCs: vascular smooth muscle cells.

## References

[1] Libby P, Buring JE, Badimon L, Hansson GK, Deanfield J, Bittencourt MS (2019). Atherosclerosis. Nat Rev Dis Primers.

[2] Einarson TR, Acs A, Ludwig C, Panton UH (2018). Prevalence of cardiovascular disease in type 2 diabetes: a systematic literature review of scientific evidence from across the world in 2007-2017. Cardiovasc Diabetol.

[3] Lusis AJ (2000). Atherosclerosis. Nature.

[4] Wang D, Yang Y, Lei Y, Tzvetkov NT, Liu X, Yeung AWK (2019). Targeting foam cell formation in atherosclerosis: therapeutic potential of natural products. Pharmacol Rev.

[5] Abdul-Ghani M, Del Prato S, Chilton R, DeFronzo RA (2016). SGLT2 inhibitors and cardiovascular risk: lessons learned from the EMPA-REG OUTCOME study. Diabetes Care.

[6] Gore MO, McGuire DK, Lingvay I, Rosenstock J (2015). Predicting cardiovascular risk in type 2 diabetes: the heterogeneity challenges. Curr Cardiol Rep.

[7] Feng X, Sureda A, Jafari S, Memariani Z, Tewari D, Annunziata G (2019). Berberine in cardiovascular and metabolic diseases: from mechanisms to therapeutics. Theranostics.

[8] Scheen AJ (2015). Pharmacodynamics, efficacy and safety of sodium-glucose co-transporter type 2 (SGLT2) inhibitors for the treatment of type 2 diabetes mellitus. Drugs.

[9] Zinman B, Wanner C, Lachin JM, Fitchett D, Bluhmki E, Hantel S (2015). Empagliflozin, cardiovascular outcomes, and mortality in type 2 diabetes. N Engl J Med.

[10] Nauck MA (2014). Update on developments with SGLT2 inhibitors in the management of type 2 diabetes. Drug Des Devel Ther.

[11] Lopaschuk GD, Verma S (2020). Mechanisms of cardiovascular benefits of sodium glucose co-transporter 2 (SGLT2) inhibitors: a state-of-the-art review. JACC Basic Transl Sci.

[12] Lambers Heerspink HJ, de Zeeuw D, Wie L, Leslie B, List J (2013). Dapagliflozin a glucose-regulating drug with diuretic properties in subjects with type 2 diabetes. Diabetes Obes Metab.

[13] Abdul-Ghani MA, Norton L, Defronzo RA (2011). Role of sodium-glucose cotransporter 2 (SGLT 2) inhibitors in the treatment of type 2 diabetes. Endocr Rev.

[14] Ferrannini E, Solini A (2012). SGLT2 inhibition in diabetes mellitus: rationale and clinical prospects. Nat Rev Endocrinol.

[15] Vallon V, Thomson SC (2017). Targeting renal glucose reabsorption to treat hyperglycaemia: the pleiotropic effects of SGLT2 inhibition. Diabetologia.

[16] Ehrenkranz JR, Lewis NG, Kahn CR, Roth J (2005). Phlorizin: a review. Diabetes Metab Res Rev.

[17] Oku A, Ueta K, Arakawa K, Ishihara T, Nawano M, Kuronuma Y (1999). T-1095, an inhibitor of renal Na+-glucose cotransporters, may provide a novel approach to treating diabetes. Diabetes.

[18] Meng W, Ellsworth BA, Nirschl AA, McCann PJ, Patel M, Girotra RN (2008). Discovery of dapagliflozin: a potent, selective renal sodium-dependent glucose cotransporter 2 (SGLT2) inhibitor for the treatment of type 2 diabetes. J Med Chem.

[19] Nomura S, Sakamaki S, Hongu M, Kawanishi E, Koga Y, Sakamoto T (2010). Discovery of canagliflozin, a novel C-glucoside with thiophene ring, as sodium-dependent glucose cotransporter 2 inhibitor for the treatment of type 2 diabetes mellitus. J Med Chem.

[20] Grempler R, Thomas L, Eckhardt M, Himmelsbach F, Sauer A, Sharp DE (2012). Empagliflozin, a novel selective sodium glucose cotransporter-2 (SGLT-2) inhibitor: characterisation and comparison with other SGLT-2 inhibitors. Diabetes Obes Metab.

[21] Rabizadeh S, Nakhjavani M, Esteghamati A (2019). Cardiovascular and renal benefits of SGLT2 inhibitors: a narrative review. Int J Endocrinol Metab.

[22] Bolinder J, Ljunggren O, Kullberg J, Johansson L, Wilding J, Langkilde AM (2012). Effects of dapagliflozin on body weight, total fat mass, and regional adipose tissue distribution in patients with type 2 diabetes mellitus with inadequate glycemic control on metformin. J Clin Endocrinol Metab.

[23] Ni L, Yuan C, Chen G, Zhang C, Wu X (2020). SGLT2i: beyond the glucose-lowering effect. Cardiovasc Diabetol.

[24] Fediuk DJ, Nucci G, Dawra VK, Cutler DL, Amin NB, Terra SG (2020). Overview of the clinical pharmacology of ertugliflozin, a novel sodium-glucose cotransporter 2 (SGLT2) inhibitor. Clin Pharmacokinet.

[25] Poole RM, Dungo RT (2014). Ipragliflozin: first global approval. Drugs.

[26] Poole RM, Prossler JE (2014). Tofogliflozin: first global approval. Drugs.

[27] Markham A, Elkinson S (2014). Luseogliflozin: first global approval. Drugs.

[28] Wanner C, Inzucchi SE, Lachin JM, Fitchett D, von Eynatten M, Mattheus M (2016). Empagliflozin and progression of kidney disease in type 2 diabetes. N Engl J Med.

[29] Packer M, Butler J, Filippatos GS, Jamal W, Salsali A, Schnee J (2019). Evaluation of the effect of sodium-glucose co-transporter 2 inhibition with empagliflozin on morbidity and mortality of patients with chronic heart failure and a reduced ejection fraction: rationale for and design of the EMPEROR-Reduced trial. Eur J Heart Fail.

[30] Packer M, Anker SD, Butler J, Filippatos G, Pocock SJ, Carson P (2020). Cardiovascular and renal outcomes with empagliflozin in heart failure. N Engl J Med.

[31] Butler J, Anker SD, Filippatos G, Khan MS, Ferreira JP, Pocock SJ (2021). Empagliflozin and health-related quality of life outcomes in patients with heart failure with reduced ejection fraction: the EMPEROR-Reduced trial. Eur Heart J.

[32] Packer M, Anker SD, Butler J, Filippatos G, Ferreira JP, Pocock SJ (2021). Influence of neprilysin inhibition on the efficacy and safety of empagliflozin in patients with chronic heart failure and a reduced ejection fraction: the EMPEROR-Reduced trial. European Heart Journal.

[33] Neal B, Perkovic V, Mahaffey KW, de Zeeuw D, Fulcher G, Erondu N (2017). Canagliflozin and cardiovascular and renal events in type 2 diabetes. N Engl J Med.

[34] Cannon CP, Perkovic V, Agarwal R, Baldassarre J, Bakris G, Charytan DM (2020). Evaluating the effects of canagliflozin on cardiovascular and renal events in patients with type 2 diabetes mellitus and chronic kidney disease according to baseline HbA1c, including those with HbA1c <7%: results from the CREDENCE trial. Circulation.

[35] Perkovic V, Jardine MJ, Neal B, Bompoint S, Heerspink HJL, Charytan DM (2019). Canagliflozin and renal outcomes in type 2 diabetes and nephropathy. N Engl J Med.

[36] Wiviott SD, Raz I, Bonaca MP, Mosenzon O, Kato ET, Cahn A (2019). Dapagliflozin and cardiovascular outcomes in type 2 diabetes. N Engl J Med.

[37] Kato ET, Silverman MG, Mosenzon O, Zelniker TA, Cahn A, Furtado RHM (2019). Effect of dapagliflozin on heart failure and mortality in type 2 diabetes mellitus. Circulation.

[38] McMurray JJV, Solomon SD, Inzucchi SE, Kober L, Kosiborod MN, Martinez FA (2019). Dapagliflozin in patients with heart failure and reduced ejection fraction. N Engl J Med.

[39] Docherty KF, Jhund PS, Inzucchi SE, Kober L, Kosiborod MN, Martinez FA (2020). Effects of dapagliflozin in DAPA-HF according to background heart failure therapy. Eur Heart J.

[40] Cannon CP, Pratley R, Dagogo-Jack S, Mancuso J, Huyck S, Masiukiewicz U (2020). Cardiovascular outcomes with ertugliflozin in type 2 diabetes. N Engl J Med.

[41] Shen Y, Zhou J, Shi L, Nauman E, Katzmarzyk PT, Price-Haywood EG (2020). Effectiveness of sodium-glucose co-transporter-2 inhibitors on ischaemic heart disease. Diabetes Obes Metab.

[42] Prattichizzo F, La Sala L, Ryden L, Marx N, Ferrini M, Valensi P (2019). Glucose-lowering therapies in patients with type 2 diabetes and cardiovascular diseases. Eur J Prev Cardiol.

[43] Kosiborod M, Birkeland KI, Cavender MA, Fu AZ, Wilding JP, Khunti K (2018). Rates of myocardial infarction and stroke in patients initiating treatment with SGLT2-inhibitors versus other glucose-lowering agents in real-world clinical practice: Results from the CVD-REAL study. Diabetes Obes Metab.

[44] Kosiborod M, Lam CSP, Kohsaka S, Kim DJ, Karasik A, Shaw J (2018). Cardiovascular Events Associated With SGLT-2 Inhibitors Versus Other Glucose-Lowering Drugs: The CVD-REAL 2 Study. J Am Coll Cardiol.

[45] Ghosh-Swaby OR, Goodman SG, Leiter LA, Cheng A, Connelly KA, Fitchett D (2020). Glucose-lowering drugs or strategies, atherosclerotic cardiovascular events, and heart failure in people with or at risk of type 2 diabetes: an updated systematic review and meta-analysis of randomised cardiovascular outcome trials. Lancet Diabetes Endocrinol.

[46] Zelniker TA, Wiviott SD, Raz I, Im K, Goodrich EL, Bonaca MP (2019). SGLT2 inhibitors for primary and secondary prevention of cardiovascular and renal outcomes in type 2 diabetes: a systematic review and meta-analysis of cardiovascular outcome trials. The Lancet.

[47] Katakami N, Mita T, Yoshii H, Shiraiwa T, Yasuda T, Okada Y (2020). Tofogliflozin does not delay progression of carotid atherosclerosis in patients with type 2 diabetes: a prospective, randomized, open-label, parallel-group comparative study. Cardiovasc Diabetol.

[48] Katakami N, Mita T, Yoshii H, Shiraiwa T, Yasuda T, Okada Y (2017). Rationale, Design, and Baseline Characteristics of the Utopia Trial for Preventing Diabetic Atherosclerosis Using an SGLT2 inhibitor: a prospective, randomized, open-label, parallel-group comparative study. Diabetes Ther.

[49] Katakami N, Mita T, Yoshii H, Shiraiwa T, Yasuda T, Okada Y (2021). Effect of tofogliflozin on arterial stiffness in patients with type 2 diabetes: prespecified sub-analysis of the prospective, randomized, open-label, parallel-group comparative UTOPIA trial. Cardiovasc Diabetol.

[50] Nasiri-Ansari N, Dimitriadis GK, Agrogiannis G, Perrea D, Kostakis ID, Kaltsas G (2018). Canagliflozin attenuates the progression of atherosclerosis and inflammation process in APOE knockout mice. Cardiovasc Diabetol.

[51] Day EA, Ford RJ, Lu JH, Lu R, Lundenberg L, Desjardins EM (2020). The SGLT2 inhibitor canagliflozin suppresses lipid synthesis and interleukin-1 beta in ApoE deficient mice. Biochem J.

[52] Nakatsu Y, Kokubo H, Bumdelger B, Yoshizumi M, Yamamotoya T, Matsunaga Y (2017). The SGLT2 inhibitor luseogliflozin rapidly normalizes aortic mRNA levels of inflammation-related but not lipid-metabolism-related genes and suppresses atherosclerosis in diabetic ApoE KO mice. Int J Mol Sci.

[53] Mori Y, Terasaki M, Hiromura M, Saito T, Kushima H, Koshibu M (2019). Luseogliflozin attenuates neointimal hyperplasia after wire injury in high-fat diet-fed mice via inhibition of perivascular adipose tissue remodeling. Cardiovasc Diabetol.

[54] Mori K, Tsuchiya K, Nakamura S, Miyachi Y, Shiba K, Ogawa Y (2019). Ipragliflozin-induced adipose expansion inhibits cuff-induced vascular remodeling in mice. Cardiovasc Diabetol.

[55] Ganbaatar B, Fukuda D, Shinohara M, Yagi S, Kusunose K, Yamada H (2020). Empagliflozin ameliorates endothelial dysfunction and suppresses atherogenesis in diabetic apolipoprotein E-deficient mice. Eur J Pharmacol.

[56] Dimitriadis GK, Nasiri-Ansari N, Agrogiannis G, Kostakis ID, Randeva MS, Nikiteas N (2019). Empagliflozin improves primary haemodynamic parameters and attenuates the development of atherosclerosis in high fat diet fed ApoE knockout mice. Mol Cell Endocrinol.

[57] Han JH, Oh TJ, Lee G, Maeng HJ, Lee DH, Kim KM (2017). The beneficial effects of empagliflozin, an SGLT2 inhibitor, on atherosclerosis in ApoE (-/-) mice fed a western diet. Diabetologia.

[58] Ortega R, Collado A, Selles F, Gonzalez-Navarro H, Sanz MJ, Real JT (2019). SGLT-2 (sodium-glucose cotransporter 2) inhibition reduces Ang II (Angiotensin II)-induced dissecting abdominal aortic aneurysm in ApoE (Apolipoprotein E) knockout mice. Arterioscler Thromb Vasc Biol.

[59] Liu Y, Xu J, Wu M, Xu B, Kang L (2021). Empagliflozin protects against atherosclerosis progression by modulating lipid profiles and sympathetic activity. Lipids Health Dis.

[60] Pennig J, Scherrer P, Gissler MC, Anto-Michel N, Hoppe N, Funer L (2019). Glucose lowering by SGLT2-inhibitor empagliflozin accelerates atherosclerosis regression in hyperglycemic STZ-diabetic mice. Sci Rep.

[61] Tomita I, Kume S, Sugahara S, Osawa N, Yamahara K, Yasuda-Yamahara M (2020). SGLT2 Inhibition Mediates Protection from Diabetic Kidney Disease by Promoting Ketone Body-Induced mTORC1 Inhibition. Cell metabolism.

[62] Leng W, Ouyang X, Lei X, Wu M, Chen L, Wu Q (2016). The SGLT-2 Inhibitor Dapagliflozin Has a Therapeutic Effect on Atherosclerosis in Diabetic ApoE(-/-) Mice. Mediators Inflamm.

[63] Terasaki M, Hiromura M, Mori Y, Kohashi K, Nagashima M, Kushima H (2015). Amelioration of hyperglycemia with a sodium-glucose cotransporter 2 inhibitor prevents macrophage-driven atherosclerosis through macrophage foam cell formation suppression in type 1 and type 2 diabetic mice. PLoS One.

[64] Taberner-Cortés A, Vinué Á, Herrero-Cervera A, Aguilar-Ballester M, Real JT, Burks DJ (2020). Dapagliflozin does not modulate atherosclerosis in mice with insulin resistance. Int J Mol Sci.

[65] Hurtubise J, McLellan K, Durr K, Onasanya O, Nwabuko D, Ndisang JF (2016). The different facets of dyslipidemia and hypertension in atherosclerosis. Curr Atheroscler Rep.

[66] Al-Sharea A, Murphy AJ, Huggins LA, Hu Y, Goldberg IJ, Nagareddy PR (2018). SGLT2 inhibition reduces atherosclerosis by enhancing lipoprotein clearance in Ldlr(-/-) type 1 diabetic mice. Atherosclerosis.

[67] Ni L, Lee SJ, Lee JJ, Kim JS, Lee OH, Kim CK (2020). Anti-inflammatory effect for atherosclerosis progression by sodium-glucose cotransporter 2 (SGLT-2) inhibitor in a normoglycemic rabbit model. Korean Circ J.

[68] Shrikrishnapalasuriyar N, Shaikh A, Ruslan AM, Sharaf G, Udiawar M, Price DE (2020). Dapagliflozin is associated with improved glycaemic control and weight reduction at 44 months of follow-up in a secondary care diabetes clinic in the UK. Diabetes Metab Syndr.

[69] Sitia S, Tomasoni L, Atzeni F, Ambrosio G, Cordiano C, Catapano A (2010). From endothelial dysfunction to atherosclerosis. Autoimmun Rev.

[70] Gimbrone MA, García-Cardeña G (2016). Endothelial cell dysfunction and the pathobiology of atherosclerosis. Circ Res.

[71] Shigiyama F, Kumashiro N, Miyagi M, Ikehara K, Kanda E, Uchino H (2017). Effectiveness of dapagliflozin on vascular endothelial function and glycemic control in patients with early-stage type 2 diabetes mellitus: DEFENCE study. Cardiovasc Diabetol.

[72] Zainordin NA, Hatta S, Mohamed Shah FZ, Rahman TA, Ismail N, Ismail Z (2020). Effects of dapagliflozin on endothelial dysfunction in type 2 diabetes with established ischemic heart disease (EDIFIED). J Endocr Soc.

[73] Solini A, Giannini L, Seghieri M, Vitolo E, Taddei S, Ghiadoni L (2017). Dapagliflozin acutely improves endothelial dysfunction, reduces aortic stiffness and renal resistive index in type 2 diabetic patients: a pilot study. Cardiovasc Diabetol.

[74] Park SH, Farooq MA, Gaertner S, Bruckert C, Qureshi AW, Lee HH (2020). Empagliflozin improved systolic blood pressure, endothelial dysfunction and heart remodeling in the metabolic syndrome ZSF1 rat. Cardiovasc Diabetol.

[75] Aroor AR, Das NA, Carpenter AJ, Habibi J, Jia G, Ramirez-Perez FI (2018). Glycemic control by the SGLT2 inhibitor empagliflozin decreases aortic stiffness, renal resistivity index and kidney injury. Cardiovasc Diabetol.

[76] Khemais-Benkhiat S, Belcastro E, Idris-Khodja N, Park SH, Amoura L, Abbas M (2020). Angiotensin II-induced redox-sensitive SGLT1 and 2 expression promotes high glucose-induced endothelial cell senescence. J Cell Mol Med.

[77] Lee DM, Battson ML, Jarrell DK, Hou S, Ecton KE, Weir TL (2018). SGLT2 inhibition via dapagliflozin improves generalized vascular dysfunction and alters the gut microbiota in type 2 diabetic mice. Cardiovasc Diabetol.

[78] Cappetta D, De Angelis A, Ciuffreda LP, Coppini R, Cozzolino A, Miccichè A (2020). Amelioration of diastolic dysfunction by dapagliflozin in a non-diabetic model involves coronary endothelium. Pharmacological Research. 2020; 157. doi: 10.1016/j.phrs.

[79] Gaspari T, Spizzo I, Liu H, Hu Y, Simpson RW, Widdop RE (2018). Dapagliflozin attenuates human vascular endothelial cell activation and induces vasorelaxation: A potential mechanism for inhibition of atherogenesis. Diab Vasc Dis Res.

[80] Tahara A, Takasu T, Yokono M, Imamura M, Kurosaki E (2017). Characterization and comparison of SGLT2 inhibitors: Part 3. Effects on diabetic complications in type 2 diabetic mice. Eur J Pharmacol.

[81] Beckman JA, Creager MA, Libby P (2002). Diabetes and atherosclerosis: epidemiology, pathophysiology, and management. JAMA.

[82] Creager MA, Luscher TF, Cosentino F, Beckman JA (2003). Diabetes and vascular disease: pathophysiology, clinical consequences, and medical therapy: Part I. Circulation.

[83] Behnammanesh G, Durante GL, Khanna YP, Peyton KJ, Durante W (2020). Canagliflozin inhibits vascular smooth muscle cell proliferation and migration: Role of heme oxygenase-1. Redox Biol.

[84] Takahashi H, Nomiyama T, Terawaki Y, Horikawa T, Kawanami T, Hamaguchi Y (2019). Combined treatment with DPP-4 inhibitor linagliptin and SGLT2 inhibitor empagliflozin attenuates neointima formation after vascular injury in diabetic mice. Biochem Biophys Rep.

[85] Adingupu DD, Gopel SO, Gronros J, Behrendt M, Sotak M, Miliotis T (2019). SGLT2 inhibition with empagliflozin improves coronary microvascular function and cardiac contractility in prediabetic ob/ob(-/-) mice. Cardiovasc Diabetol.

[86] Tian K, Xu Y, Sahebkar A, Xu S (2020). CD36 in atherosclerosis: pathophysiological mechanisms and therapeutic implications. Curr Atheroscler Rep.

[87] Lin B, Koibuchi N, Hasegawa Y, Sueta D, Toyama K, Uekawa K (2014). Glycemic control with empagliflozin, a novel selective SGLT2 inhibitor, ameliorates cardiovascular injury and cognitive dysfunction in obese and type 2 diabetic mice. Cardiovasc Diabetol.

[88] Xu L, Nagata N, Nagashimada M, Zhuge F, Ni Y, Chen G (2017). SGLT2 Inhibition by Empagliflozin Promotes Fat Utilization and Browning and Attenuates Inflammation and Insulin Resistance by Polarizing M2 Macrophages in Diet-induced Obese Mice. EBioMedicine.

[89] Koyani CN, Plastira I, Sourij H, Hallstrom S, Schmidt A, Rainer PP (2020). Empagliflozin protects heart from inflammation and energy depletion via AMPK activation. Pharmacol Res.

[90] Mancini SJ, Boyd D, Katwan OJ, Strembitska A, Almabrouk TA, Kennedy S (2018). Canagliflozin inhibits interleukin-1beta-stimulated cytokine and chemokine secretion in vascular endothelial cells by AMP-activated protein kinase-dependent and -independent mechanisms. Sci Rep.

[91] Lee TM, Chang NC, Lin SZ (2017). Dapagliflozin, a selective SGLT2 Inhibitor, attenuated cardiac fibrosis by regulating the macrophage polarization via STAT3 signaling in infarcted rat hearts. Free Radic Biol Med.

[92] Wu MD, Atkinson TM, Lindner JR (2017). Platelets and von Willebrand factor in atherogenesis. Blood.

[93] Kraakman MJ, Lee MK, Al-Sharea A, Dragoljevic D, Barrett TJ, Montenont E (2017). Neutrophil-derived S100 calcium-binding proteins A8/A9 promote reticulated thrombocytosis and atherogenesis in diabetes. J Clin Invest.

[94] Spigoni V, Fantuzzi F, Carubbi C, Pozzi G, Masselli E, Gobbi G (2020). Sodium-glucose cotransporter 2 inhibitors antagonize lipotoxicity in human myeloid angiogenic cells and ADP-dependent activation in human platelets: potential relevance to prevention of cardiovascular events. Cardiovasc Diabetol.

[95] Sakurai S, Jojima T, Iijima T, Tomaru T, Usui I, Aso Y (2020). Empagliflozin decreases the plasma concentration of plasminogen activator inhibitor-1 (PAI-1) in patients with type 2 diabetes: Association with improvement of fibrinolysis. J Diabetes Complications.

[96] Oelze M, Kroller-Schon S, Welschof P, Jansen T, Hausding M, Mikhed Y (2014). The sodium-glucose co-transporter 2 inhibitor empagliflozin improves diabetes-induced vascular dysfunction in the streptozotocin diabetes rat model by interfering with oxidative stress and glucotoxicity. PLoS One.

[97] Rahadian A, Fukuda D, Salim HM, Yagi S, Kusunose K, Yamada H (2020). Canagliflozin prevents diabetes-induced vascular dysfunction in ApoE-deficient mice. Journal of Atherosclerosis and Thrombosis.

[98] Sayour AA, Korkmaz-Icoz S, Loganathan S, Ruppert M, Sayour VN, Olah A (2019). Acute canagliflozin treatment protects against *in vivo* myocardial ischemia-reperfusion injury in non-diabetic male rats and enhances endothelium-dependent vasorelaxation. J Transl Med.

[99] Steven S, Oelze M, Hanf A, Kroller-Schon S, Kashani F, Roohani S (2017). The SGLT2 inhibitor empagliflozin improves the primary diabetic complications in ZDF rats. Redox Biol.

[100] Kolijn D, Pabel S, Tian Y, Lodi M, Herwig M, Carrizzo A (2020). Empagliflozin improves endothelial and cardiomyocyte function in human heart failure with preserved ejection fraction via reduced pro-inflammatory-oxidative pathways and protein kinase Galpha oxidation. Cardiovasc Res.

[101] Salim HM, Fukuda D, Yagi S, Soeki T, Shimabukuro M, Sata M (2016). Glycemic control with ipragliflozin, a novel selective SGLT2 inhibitor, ameliorated endothelial dysfunction in streptozotocin-induced diabetic mouse. Front Cardiovasc Med.

[102] Uthman L, Homayr A, Juni RP, Spin EL, Kerindongo R, Boomsma M (2019). Empagliflozin and dapagliflozin reduce ROS generation and restore NO bioavailability in tumor necrosis factor alpha-stimulated human coronary arterial endothelial cells. Cell Physiol Biochem.

[103] El-Daly M, Pulakazhi Venu VK, Saifeddine M, Mihara K, Kang S, Fedak PWM (2018). Hyperglycaemic impairment of PAR2-mediated vasodilation: Prevention by inhibition of aortic endothelial sodium-glucose-co-Transporter-2 and minimizing oxidative stress. Vascul Pharmacol.

[104] Garvey WT, Van Gaal L, Leiter LA, Vijapurkar U, List J, Cuddihy R (2018). Effects of canagliflozin versus glimepiride on adipokines and inflammatory biomarkers in type 2 diabetes. Metabolism.

[105] Heerspink HJL, Perco P, Mulder S, Leierer J, Hansen MK, Heinzel A (2019). Canagliflozin reduces inflammation and fibrosis biomarkers: a potential mechanism of action for beneficial effects of SGLT2 inhibitors in diabetic kidney disease. Diabetologia.

[106] Iannantuoni F, M de Marañon A, Diaz-Morales N, Falcon R, Bañuls C, Abad-Jimenez Z (2019). The SGLT2 Inhibitor Empagliflozin Ameliorates the Inflammatory Profile in Type 2 Diabetic Patients and Promotes an Antioxidant Response in Leukocytes. J Clin Med.

[107] Cornelissen A, Guo L, Sakamoto A, Virmani R, Finn AV (2019). New insights into the role of iron in inflammation and atherosclerosis. EBioMedicine.

[108] Kempf T, Wollert KC (2020). Iron and atherosclerosis: too much of a good thing can be bad. Eur Heart J.

[109] Vinchi F, Porto G, Simmelbauer A, Altamura S, Passos ST, Garbowski M (2020). Atherosclerosis is aggravated by iron overload and ameliorated by dietary and pharmacological iron restriction. Eur Heart J.

[110] You SA, Wang Q (2005). Ferritin in atherosclerosis. Clin Chim Acta.

[111] Xu S (2019). Iron and atherosclerosis: the link revisited. Trends Mol Med.

[112] Katsiki N, Mikhailidis DP (2019). Iron absorption, bone marrow fat and hematopoiesis in heart failure: Additional mechanisms of action for sodium-glucose co-transporter 2 inhibitors (SGLT2i)?. J Diabetes Complications.

[113] Ferrannini E, Murthy AC, Lee YH, Muscelli E, Weiss S, Ostroff RM (2020). Mechanisms of sodium-glucose cotransporter 2 inhibition: insights from large-scale proteomics. Diabetes Care.

[114] Ghanim H, Abuaysheh S, Hejna J, Green K, Batra M, Makdissi A (2020). Dapagliflozin Suppresses Hepcidin And Increases Erythropoiesis. J Clin Endocrinol Metab.

[115] Liao X, Sluimer JC, Wang Y, Subramanian M, Brown K, Pattison JS (2012). Macrophage autophagy plays a protective role in advanced atherosclerosis. Cell Metab.

[116] Xu C, Wang W, Zhong J, Lei F, Xu N, Zhang Y (2018). Canagliflozin exerts anti-inflammatory effects by inhibiting intracellular glucose metabolism and promoting autophagy in immune cells. Biochem Pharmacol.

[117] Umino H, Hasegawa K, Minakuchi H, Muraoka H, Kawaguchi T, Kanda T (2018). High Basolateral Glucose Increases Sodium-Glucose Cotransporter 2 and Reduces Sirtuin-1 in Renal Tubules through Glucose Transporter-2 Detection. Sci Rep.

[118] Aragon-Herrera A, Feijoo-Bandin S, Otero Santiago M, Barral L, Campos-Toimil M, Gil-Longo J (2019). Empagliflozin reduces the levels of CD36 and cardiotoxic lipids while improving autophagy in the hearts of Zucker diabetic fatty rats. Biochem Pharmacol.

[119] Mizuno M, Kuno A, Yano T, Miki T, Oshima H, Sato T (2018). Empagliflozin normalizes the size and number of mitochondria and prevents reduction in mitochondrial size after myocardial infarction in diabetic hearts. Physiol Rep.

[120] Wang CY, Chen CC, Lin MH, Su HT, Ho MY, Yeh JK (2020). TLR9 binding to beclin 1 and mitochondrial SIRT3 by a sodium-glucose co-transporter 2 inhibitor protects the heart from doxorubicin toxicity. Biology (Basel).

[121] Li H, Shin SE, Seo MS, An JR, Choi IW, Jung WK (2018). The anti-diabetic drug dapagliflozin induces vasodilation via activation of PKG and Kv channels. Life Sci.

[122] Baartscheer A, Schumacher CA, Wust RC, Fiolet JW, Stienen GJ, Coronel R (2017). Empagliflozin decreases myocardial cytoplasmic Na(+) through inhibition of the cardiac Na(+)/H(+) exchanger in rats and rabbits. Diabetologia.

[123] Chung YJ, Park KC, Tokar S, Eykyn TR, Fuller W, Pavlovic D (2020). Off-target effects of SGLT2 blockers: empagliflozin does not inhibit Na+/H+ exchanger-1 or lower [Na+]i in the heart. Cardiovasc Res.

[124] Prattichizzo F, De Nigris V, Micheloni S, La Sala L, Ceriello A (2018). Increases in circulating levels of ketone bodies and cardiovascular protection with SGLT2 inhibitors: Is low-grade inflammation the neglected component?. Diabetes Obes Metab.

[125] Ferrannini E, Mark M, Mayoux E (2016). CV Protection in the EMPA-REG OUTCOME Trial: A "Thrifty Substrate" Hypothesis. Diabetes Care.

[126] Verma S, Rawat S, Ho KL, Wagg CS, Zhang L, Teoh H (2018). Empagliflozin increases cardiac energy production in diabetes: novel translational insights into the heart failure benefits of SGLT2 inhibitors. JACC Basic Transl Sci.

[127] Ferrannini E, Baldi S, Frascerra S, Astiarraga B, Heise T, Bizzotto R (2016). Shift to fatty substrate utilization in response to sodium-glucose cotransporter 2 inhibition in subjects without diabetes and patients with type 2 diabetes. Diabetes.

[128] Kim SR, Lee SG, Kim SH, Kim JH, Choi E, Cho W (2020). SGLT2 inhibition modulates NLRP3 inflammasome activity via ketones and insulin in diabetes with cardiovascular disease. Nat Commun.

[129] Rajeev SP, Cuthbertson DJ, Wilding JPH (2016). Energy balance and metabolic changes with sodium-glucose co-transporter 2 inhibition. Diabetes Obes Metab.

[130] Storgaard H, Gluud LL, Bennett C, Grondahl MF, Christensen MB, Knop FK (2016). Benefits and harms of sodium-glucose co-transporter 2 inhibitors in patients with type 2 diabetes: a systematic review and meta-analysis. PLoS One.

[131] Cai X, Yang W, Gao X, Chen Y, Zhou L, Zhang S (2018). The association between the dosage of SGLT2 inhibitor and weight reduction in type 2 diabetes patients: a meta-analysis. Obesity (Silver Spring).

[132] Heerspink HJL, Perkins BA, Fitchett DH, Husain M, Cherney DZI (2016). Sodium glucose cotransporter 2 inhibitors in the treatment of diabetes mellitus: cardiovascular and kidney effects, Potential Mechanisms, and Clinical Applications. Circulation.

[133] Lytvyn Y, Bjornstad P, Udell JA, Lovshin JA, Cherney DZI (2017). Sodium glucose cotransporter-2 inhibition in heart failure: potential mechanisms, clinical applications, and summary of clinical trials. Circulation.

[134] Tikkanen I, Narko K, Zeller C, Green A, Salsali A, Broedl UC (2015). Empagliflozin reduces blood pressure in patients with type 2 diabetes and hypertension. Diabetes Care.

[135] Wilcox CS (2020). Antihypertensive and renal mechanisms of SGLT2 (sodium-glucose linked transporter 2) inhibitors. Hypertension.

[136] Davies MJ, Trujillo A, Vijapurkar U, Damaraju CV, Meininger G (2015). Effect of canagliflozin on serum uric acid in patients with type 2 diabetes mellitus. Diabetes Obes Metab.

[137] Zhao Y, Xu L, Tian D, Xia P, Zheng H, Wang L (2018). Effects of sodium-glucose co-transporter 2 (SGLT2) inhibitors on serum uric acid level: A meta-analysis of randomized controlled trials. Diabetes Obes Metab.

[138] Hao Z, Huang X, Shao H, Tian F (2018). Effects of dapagliflozin on serum uric acid levels in hospitalized type 2 diabetic patients with inadequate glycemic control: a randomized controlled trial. Ther Clin Risk Manag.

[139] Braga TT, Forni MF, Correa-Costa M, Ramos RN, Barbuto JA, Branco P (2017). Soluble uric acid activates the NLRP3 inflammasome. Sci Rep.

[140] De Nardo D, Latz E (2011). NLRP3 inflammasomes link inflammation and metabolic disease. Trends Immunol.

[141] Duewell P, Kono H, Rayner KJ, Sirois CM, Vladimer G, Bauernfeind FG (2010). NLRP3 inflammasomes are required for atherogenesis and activated by cholesterol crystals. Nature.

[142] Van Tassell BW, Toldo S, Mezzaroma E, Abbate A (2013). Targeting interleukin-1 in heart disease. Circulation.

[143] Guo H, Callaway JB, Ting JP (2015). Inflammasomes: mechanism of action, role in disease, and therapeutics. Nat Med.

[144] Byrne NJ, Matsumura N, Maayah ZH, Ferdaoussi M, Takahara S, Darwesh AM (2020). Empagliflozin blunts worsening cardiac dysfunction associated with reduced NLRP3 (nucleotide-binding domain-like receptor protein 3) inflammasome activation in heart failure. Circulation: Heart Failure.

[145] Ye Y, Bajaj M, Yang HC, Perez-Polo JR, Birnbaum Y (2017). SGLT-2 inhibition with dapagliflozin reduces the activation of the Nlrp3/ASC inflammasome and attenuates the development of diabetic cardiomyopathy in mice with type 2 diabetes. further augmentation of the effects with saxagliptin, a DPP4 inhibitor. Cardiovasc Drugs Ther.

[146] Chen H, Tran D, Yang HC, Nylander S, Birnbaum Y, Ye Y (2020). Dapagliflozin and ticagrelor have additive effects on the attenuation of the activation of the NLRP3 inflammasome and the progression of diabetic cardiomyopathy: an AMPK-mTOR interplay. Cardiovasc Drugs Ther.

[147] Isermann B, Bierhaus A, Humpert PM, Rudofsky G, Chavakis T, Ritzel R (2004). [AGE-RAGE: a hypothesis or a mechanism?]. Herz.

[148] Longato E, Di Camillo B, Sparacino G, Gubian L, Avogaro A, Fadini GP (2020). Cardiovascular outcomes of type 2 diabetic patients treated with SGLT-2 inhibitors versus GLP-1 receptor agonists in real-life. BMJ Open Diabetes Res Care.

[149] Petrie MC, Verma S, Docherty KF, Inzucchi SE, Anand I, Belohlavek J (2020). Effect of dapagliflozin on worsening heart failure and cardiovascular death in patients with heart failure with and without diabetes. JAMA.

[150] Li W, Yu K, Sun S (2020). Novel oral hypoglycemic agents SGLT-2 inhibitors: cardiovascular benefits and potential mechanisms. Pharmazie.

[151] Cowie MR, Fisher M (2020). SGLT2 inhibitors: mechanisms of cardiovascular benefit beyond glycaemic control. Nat Rev Cardiol.

[152] Dardano A, Miccoli R, Bianchi C, Daniele G, Del Prato S (2020). Invited review. Series: Implications of the recent CVOTs in type 2 diabetes: Which patients for GLP-1RA or SGLT-2 inhibitor?. Diabetes Res Clin Pract.

[153] Kang Y, Zhan F, He M, Liu Z, Song X (2020). Anti-inflammatory effects of sodium-glucose co-transporter 2 inhibitors on atherosclerosis. Vascular Pharmacology.

[154] Paul SK, Bhatt DL, Montvida O (2020). The association of amputations and peripheral artery disease in patients with type 2 diabetes mellitus receiving sodium-glucose cotransporter type-2 inhibitors: real-world study. Eur Heart J.

[155] Wang W, Zhang L, Pei X, Pan Q, Guo L (2020). Evaluation of the safety of sodium-glucose co-transporter-2 inhibitors for treating patients with type 1 diabetes. Diabetes Obes Metab.

[156] Saisho Y (2020). SGLT2 Inhibitors: the Star in the Treatment of Type 2 Diabetes?. Diseases.

