# Supplementary material for: Impact of sodium glucose cotransporter 2 (SGLT2) inhibitors on atherosclerosis: from pharmacology to pre-clinical and clinical therapeutics
Source: Theranostics. 2021 Mar 4;11(9):4502–15. doi: 10.7150/thno.54498 (PMC7977463; doi:10.7150/thno.54498)
Supplement: Supplementary file 1 — Supplementary figure. [file thnov11p4502s1.pdf]

Supplemental materials

Impact of sodium glucose cotransporter 2 (SGLT2) inhibitors on atherosclerosis: from pharmacology to pre-clinical and clinical therapeutics

Zhenghong Liu[1], Xiaoxuan Ma[1], Iqra Ilyas[1], Xueying Zheng[1], Sihui Luo[1], Peter J. Little[2,3], Danielle Kamato[3], Amirhossein Sahebka[4,5], Weiming Wu[6], Jianping Weng[1*], Suowen Xu[1*]

Figure S1

Expression of SGLT1 and SGLT2 genes in different human tissues

(A) Expression of SGLT1 gene in different tissues, (B) Expression of SGLT2 gene in different tissues accessed from http://genome.ucsc.edu/

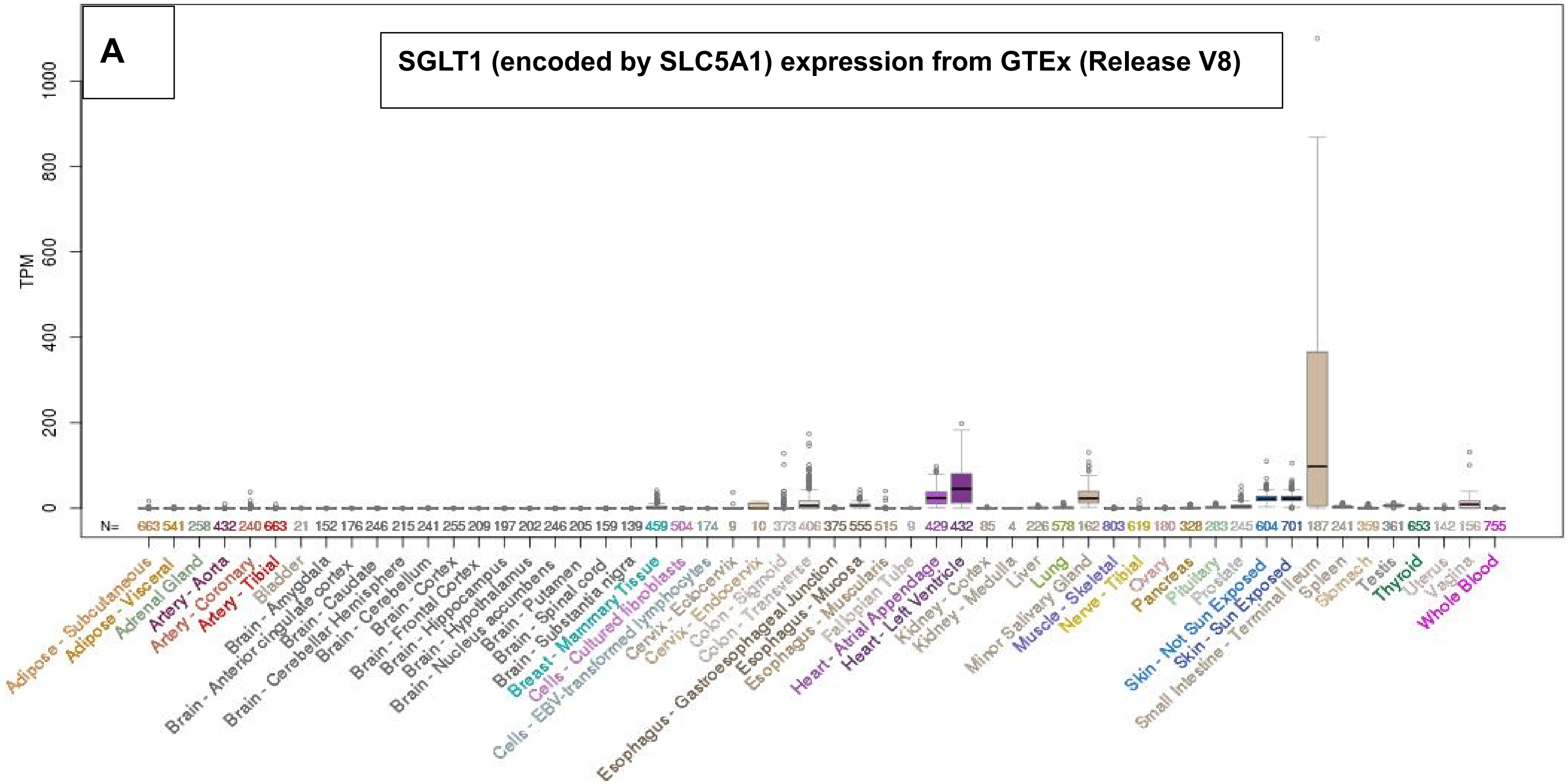

A
SGLT1 (encoded by SLC5A1) expression from GTEx (Release V8)
TPM
0
200
400
600
800
1000
N=
663 541 258 432 240 663 21 152 176 246 215 241 255 209 197 202 246 205 159 139 459 504 174 9 10 373 406 375 555 515 9 429 432 85 4 226 578 162 803 619 180 328 283 245 604 701 187 241 359 361 653 142 156 755
Adipose - Subcutaneous
Adipose - Visceral
Adrenal Gland
Artery - Aorta
Artery - Coronary
Artery - Tibial
Bladder
Brain - Amygdala
Brain - Anterior cingulate cortex
Brain - Caudate
Brain - Cerebellar Hemisphere
Brain - Cerebellum
Brain - Cortex
Brain - Frontal Cortex
Brain - Hippocampus
Brain - Hypothalamus
Brain - Nucleus accumbens
Brain - Putamen
Brain - Spinal cord
Brain - Substantia nigra
Breast - Mammary Tissue
Cells - Cultured fibroblasts
Cells - EBV-transformed lymphocytes
Cervix - Ectocervix
Cervix - Endocervix
Colon - Sigmoid
Colon - Transverse
Esophagus - Gastroesophageal Junction
Esophagus - Mucosa
Esophagus - Muscularis
Fallopian Tube
Heart - Atrial Appendage
Heart - Left Ventricle
Kidney - Cortex
Kidney - Medulla
Liver
Lung
Minor Salivary Gland
Muscle - Skeletal
Nerve - Tibial
Ovary
Pancreas
Pituitary
Prostate
Skin - Not Sun Exposed
Skin - Sun Exposed
Small Intestine - Terminal Ileum
Spleen
Stomach
Testis
Thyroid
Uterus
Vagina
Whole Blood

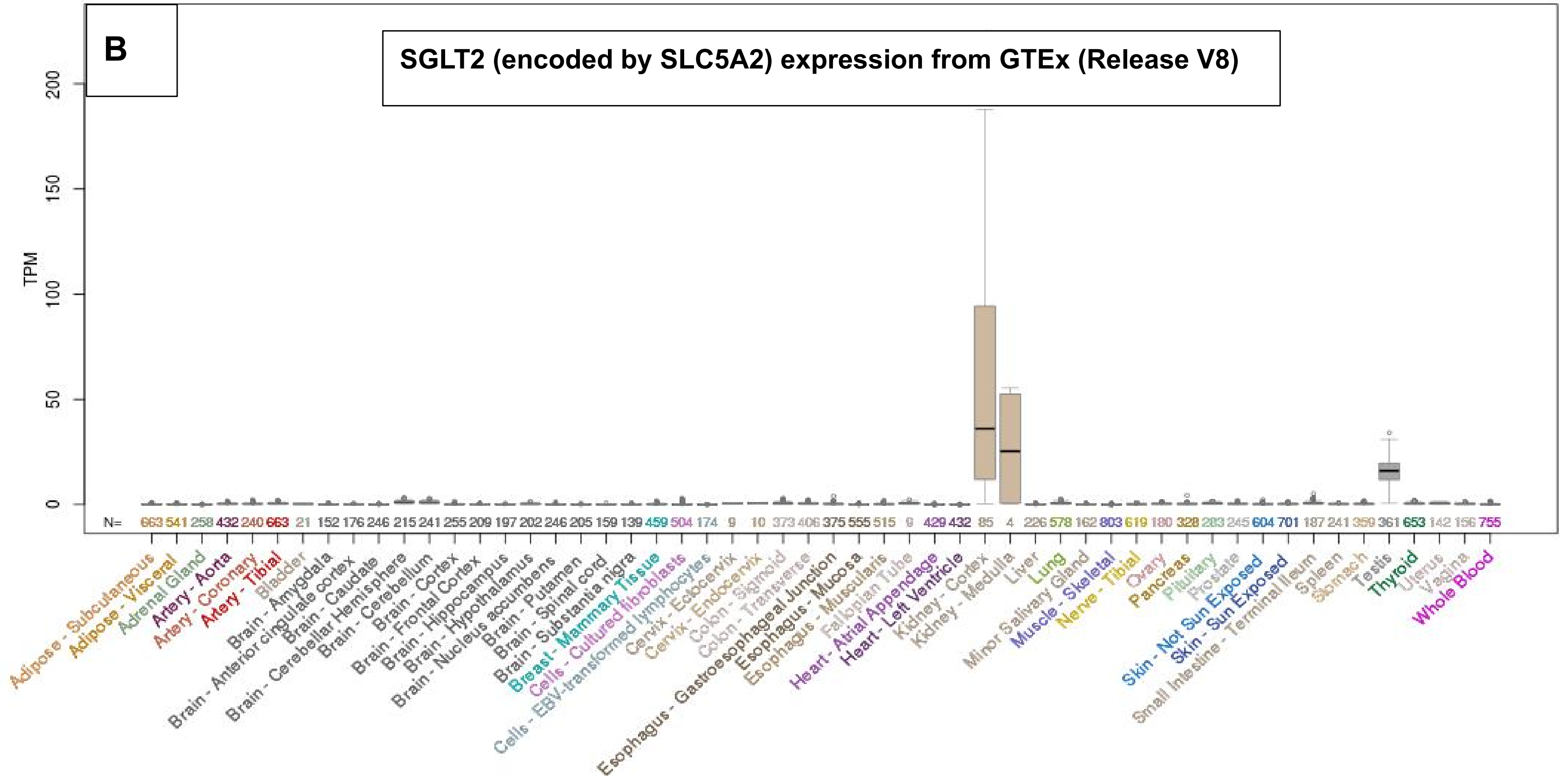
B
SGLT2 (encoded by SLC5A2) expression from GTEx (Release V8)
TPM
0
50
100
150
200
N=
663 541 258 432 240 663 21 152 176 246 215 241 255 209 197 202 246 205 159 139 459 504 174 9 10 373 406 375 555 515 9 429 432 85 4 226 578 162 803 619 180 328 283 245 604 701 187 241 359 361 653 142 156 755
Adipose - Subcutaneous
Adipose - Visceral
Adrenal Gland
Artery - Aorta
Artery - Coronary
Artery - Tibial
Bladder
Brain - Amygdala
Brain - Anterior cingulate cortex
Brain - Caudate
Brain - Cerebellar Hemisphere
Brain - Cerebellum
Brain - Cortex
Brain - Frontal Cortex
Brain - Hippocampus
Brain - Hypothalamus
Brain - Nucleus accumbens
Brain - Putamen
Brain - Spinal cord
Brain - Substantia nigra
Breast - Mammary Tissue
Cells - Cultured fibroblasts
Cells - EBV-transformed lymphocytes
Cervix - Ectocervix
Cervix - Endocervix
Colon - Sigmoid
Colon - Transverse
Esophagus - Gastroesophageal Junction
Esophagus - Mucosa
Esophagus - Muscularis
Fallopian Tube
Heart - Atrial Appendage
Heart - Left Ventricle
Kidney - Cortex
Kidney - Medulla
Liver
Lung
Minor Salivary Gland
Muscle - Skeletal
Nerve - Tibial
Ovary
Pancreas
Pituitary
Prostate
Skin - Not Sun Exposed
Skin - Sun Exposed
Small Intestine - Terminal Ileum
Spleen
Stomach
Testis
Thyroid
Uterus
Vagina
Whole Blood
